# Amphibian richness, rarity, threats, and conservation prospects across the U.S. National Park System

**DOI:** 10.1038/s44185-024-00067-1

**Published:** 2024-11-21

**Authors:** Benjamin J. LaFrance, Andrew M. Ray, Michael T. Tercek, Robert N. Fisher, Blake R. Hossack

**Affiliations:** 1 National Park Service, Greater Yellowstone Network, Bozeman, MT 59715 USA; 2 National Park Service, Southern Plains Network, Pecos, NM 87552 USA; 3 Walking Shadow Ecology, Gardiner, MT 59030 USA; 4 U.S. Geological Survey, Western Ecological Research Center, San Diego, CA 92101 USA; 5 U.S. Geological Survey, Northern Rocky Mountain Science Center, Missoula, MT 59812 USA; 6 Wildlife Biology Program, University of Montana, Missoula, MT 59812 USA

**Keywords:** Biodiversity, Conservation biology, Invasive species

## Abstract

We assessed amphibian diversity, rarity, and threats across the National Park System (U.S.A.), which covers 3.5% of the country and 12% of federal lands. At least 230 of 354 (65%) amphibian species documented in the country occur on National Park Service lands. Of species in parks, 17% are at-risk globally and 20% are uncategorized, reflecting still-widespread data deficiencies. National parks in the Northwest and Northeast had the steepest species‒area relationships. Non-native crayfishes and amphibians occur within 50 km of 60% and 25% of parks, respectively, illustrating the broad threat of non-native predators. Projected mid-century (2040–2069) changes in climatic water deficit, based on 25 climate futures, produced an expected 34% increase in dryness across all national parks in the conterminous U.S.A. Our analyses highlight the extent and regional differences in current and future threats and reveal gaps in species protection, but also reveal opportunities for targeted expansion and active management.

## Introduction

Protected areas are a fundamental underpinning for the conservation of biodiversity^1,2^. Preserved lands often serve as a proxy for reference conditions, especially for areas most affected by human alteration^3,4^. Also, because many protected areas were preserved for their distinct physical or ecological features, they often contain critical habitats or microclimates lacking in surrounding landscapes and can thus serve as climate refugia or be managed to support climate change adaptation strategies^5–7^. The importance of protected areas for conservation will likely increase, especially in areas most threatened by land use change, invasive species, and climate change^7,8^.

Protected areas managed for biodiversity include federal and state wilderness areas, national parks, and wildlife refuges. Indigenous, state or provincial, and private lands can also be managed to conserve biodiversity. Collectively, these lands contribute to a network of protected areas critical for realizing the goals ratified under the Kunming-Montreal Global Biodiversity Framework of the United Nations Convention on Biological Diversity to expand protected areas and conserve biodiversity^1,9^. The framework set a target of expanding land and water protections to 30% by 2030 (Target 3)^9^. This conservation target has received much attention, with many nations, including the United States of America (U.S.), advancing similar targets for protected areas (e.g., 30 by 30^10^). What is less clear is how nations, states, or even land management agencies should prioritize protected area expansion^10^, but careful planning is needed to ensure protected areas are placed in regions of greatest conservation importance to maximize biodiversity benefits^1^. Here, we assess amphibian diversity, rarity, and primary threats across the National Park System in the U.S. and highlight opportunities to expand this protected area network to help achieve local, national, and global conservation goals.

Lands managed by the U.S. National Park Service (NPS) represent >12% of federal lands in the U.S. and are a central component of the federal conservation network^4,11^. Although NPS lands are largely shielded from development and managed for biodiversity, the protected status of these lands does not always safeguard against population declines^12,13^. Indeed, research in national parks was central to revealing the scale and scope of amphibian declines^14^. In the 1990s, widespread declines or extirpations of several species in national parks provided some of the earliest evidence of enigmatic declines that became emblematic of the amphibian decline crisis^15–17^. Many of these declines were later linked to drought, non-native predators, and disease^14,18,19^. Still, most amphibian species in the U.S. occur in or near national parks^20^ and for many of these species, national parks contain some of the best current and future habitat e.g., ref. ^7^.

**Fig. 1:**
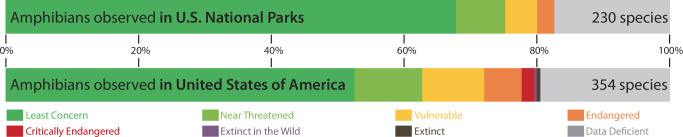
Representation of amphibian species documented on U.S. National Park Service (NPS) lands relative to all lands in the U.S.A. and categorized according to IUCN Red List status, based on a recently updated NPS dataset^20^.

A recent analysis revealed that, of the approximately 354 amphibian species documented in the U.S., 230 (65%) had been documented in parks (Fig. 1)^20^. While at-risk amphibians are under-represented in national parks — only 30% of vulnerable, 20% of endangered, and 0% of critically endangered amphibian species have been documented in parks — parks still provide critical habitat for many imperiled species, most of which are endemic to the U.S.^20^. As of 2012, the proportion of vertebrate species endemic to the U.S. was much higher for amphibians (~70%) and freshwater fishes (~68%) than for reptiles (~30%), mammals (~28%), or birds (~3%), and amphibians especially tend to have small, non-overlapping distribution ranges^21^. For instance, several salamanders occur only (e.g., *Plethodon shenandoah*, *P. jordani*) or primarily (e.g., *P. neomexicanus*) in national parks, many of which are now threatened by climate-related changes in moisture and fire regimes^22,23^.

Non-native animals, including many fishes, crayfishes, and amphibians, are a growing threat to biodiversity worldwide and have been documented in more than half of U.S. national parks^24,25^, where they may reduce the conservation value of protected lands. Non-native predators have been linked with local or regional decline of several native amphibians in national parks and other protected lands, especially in western North America^14,25,26^. For example, in the Northwest and Southwest, American Bullfrogs (*Lithobates catesbeianus*) are a primary threat to many imperiled amphibians that require semi-permanent or permanent sources of water^27,28^. Bullfrogs are strong predators and dispersers that are also associated with increased local occurrence of the emerging pathogens amphibian chytrid fungus (*Batrachochytrium dendrobatidis*) and ranaviruses^29–31^. Although there is less information compared to the threat posed by introduced amphibians and fishes, non-native crayfishes have also been introduced across much of the U.S. and globally and are often a conservation threat to amphibians and other native aquatic species^26,32^.

We used a recently updated database of amphibian occurrence for U.S. national parks^20^ to assess species-level representation, conservation status based on state and global conservation ranks, potential threats from non-native crayfishes and amphibians, and projected changes in water availability. This information can be used to prioritize conservation actions such as translocating animals to a climate refuge or safeguarding isolated populations^1,33,34^. Based on species–area relationships and representation of imperiled species, we also provide guidance on where targeted land protections or acquisitions and other conservation actions could help reduce current and future threats to species^1,35^.

## Results

As of 2023, 354 amphibian species were documented in the U.S.^20^. Of these, 52.5% have an IUCN conservation status of least concern^36^. At-risk species — those listed as vulnerable (33), endangered (20) and critically endangered (7) by the IUCN — represent 17% of all U.S. amphibian species. Another 19.5% of species were not categorized, often due to data deficiency. The IUCN lists two extinct amphibian species (*Plethodon ainsworthi*, *Lithobates fisheri*) from the U.S., neither of which were documented in national parks. Within national parks, 230 amphibian species were documented as of 2023^20^. Of these species, 69% have an IUCN status of least concern while 7% (ten vulnerable and six endangered) are listed as at-risk^36^.

**Fig. 2:**
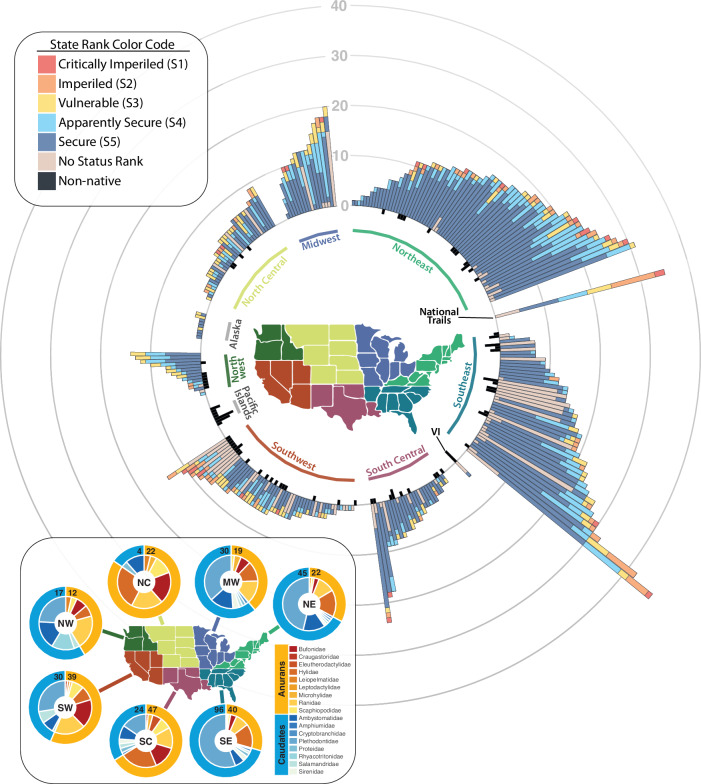
Species richness of amphibians on National Park Service (NPS) lands, based on parks and phylogenies for major regions in the U.S.A, including the Pacific Islands and the Virgin Islands (VI). In the main figure, each stacked bar represents the number of species documented per park and their NatureServe state-level conservation status. Non-native species are plotted in the negative direction in black. National trails are summarized separately because they often cross regions. The inset figure represents regional biodiversity portrayed for taxonomic orders (outer ring; anurans = frogs and toads, caudates = salamanders) and the number of species by taxonomic family (inner ring). The number of unique anuran and caudate species per region is shown as the numbers at the top of each pie chart. In the main figure, a species that occurs in several parks is represented in different bars, whereas in the inset figure, a species is represented only once per region.

Parks in the Southeast and Northeast, where salamander species outnumber those of frogs and toads, had more species than those in the western two-thirds of the country, where frogs and toads tend to outnumber salamander species (Fig. 2). Relative to the rest of the country, a greater proportion of amphibian species documented in southwestern parks are classified as near threatened, vulnerable, or endangered (24%) based on the IUCN ranks, followed by the Southeast (13%) and the Northwest (9%)^37^. Based on state ranks, a greater proportion of species in the Southwest also have no assigned conservation status (29% of records; Fig. 2).

**Fig. 3:**
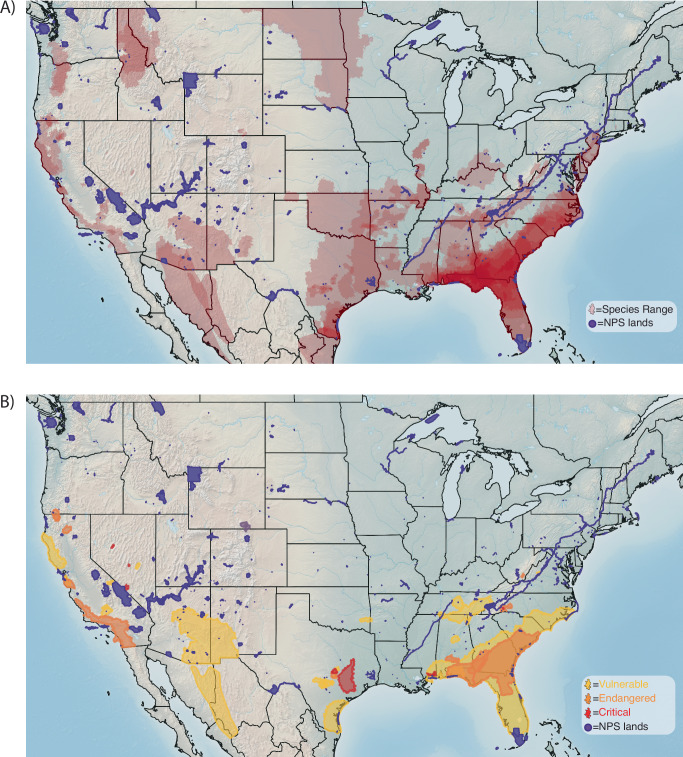
Native amphibian species as-yet undocumented on National Park Service (NPS) lands in the conterminous U.S.A. Range maps for **A** all amphibians native to the conterminous U.S.A. but as-yet undocumented on National Park Service (NPS) lands. The same map **B** but limited to species of global conservation concern (IUCN) that have not been documented on NPS lands. Alaska and all Pacific Islands are excluded because all amphibian species known in Alaska have been documented on NPS lands, and there are no amphibians native to NPS lands in the Pacific Islands. The range maps represent historical distributions of species, not necessarily current distributions.

Most species that have not been documented in national parks are in the Southeast and South Central, where species richness is greatest (Fig. 3A, B). Many of these species have small ranges, like the West Virginia Spring Salamander (*Gyrinophilus subterraneus*) and Hot Creek Toad (*Anaxyrus monfontanus*), or they are in areas with few national parks, like the Houston Toad (*Anaxyrus houstonensis*) and Apalachicola Waterdog (*Necterus moleri*). However, even some species with relatively large ranges, such as the Canadian Toad (*Anaxyrus hemiophrys*) and Strecker’s Chorus Frog (*Pseudacris streckeri*), also have not been documented in parks.

**Fig. 4:**
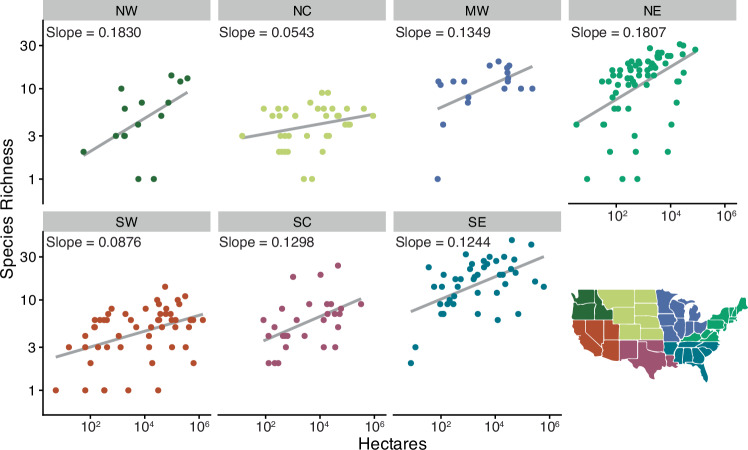
Species‒area relationships for richness of native amphibians on National Park Service (NPS) lands in seven regions that represent the conterminous U.S.A. We excluded the Pacific Islands, Caribbean Islands, and Alaska from these plots because of low richness of native species (islands) or because large areas have unsuitable climates for all but a very few amphibians (Alaska) that occur in parks in the Northwest. See Fig. 2 for definitions of region labels.

National parks in the Northwest and Northeast had the steepest species‒area relationships, indicating more rapid accumulation of species as park size increased (Fig. 4). Parks in the North Central and Southwest had the fewest species per area. The North Central had the fewest species overall, including only four salamander species documented on NPS lands (Fig. 2). Most species in the North Central region are habitat generalists with large ranges that overlap other regions. The low number of species in the southwestern parks was surprising. However, several parks in the Southwest have only one native amphibian species documented; most of these sites are small national monuments or historic sites (e.g., Cabrillo National Monument, Eugene O’Neill National Historic Site) or occur in dry, under-surveyed areas. Also, in contrast with other regions with large, montane parks (e.g., Northwest), the largest parks in the Southwest do not have the highest species richness (Fig. 4).

**Fig. 5:**
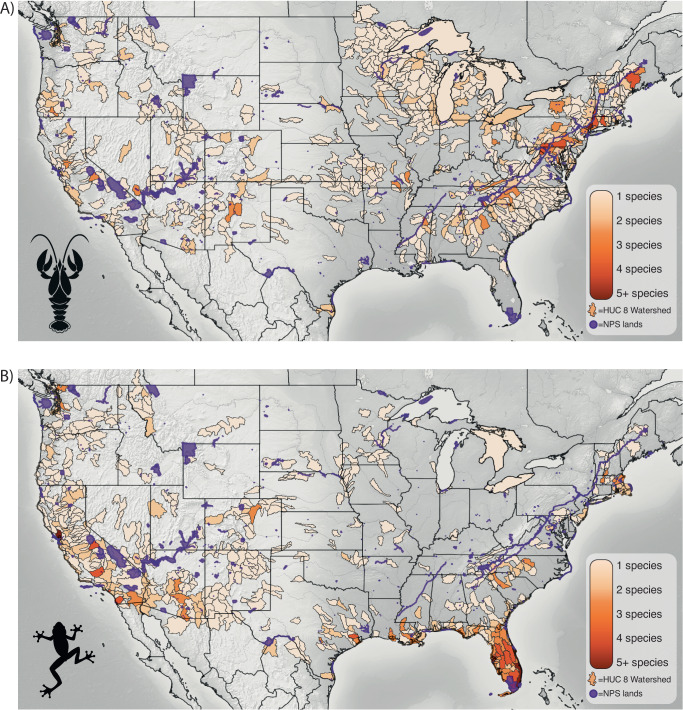
Summary of potential threats from non-native predators to native amphibians in the conterminous U.S.A. Threat is summarized as the number of non-native crayfish **A** and non-native amphibian **B** species reported from hydrologic unit code (HUC) 8 watersheds that overlap with the National Park Service (NPS) lands (purple). Species records were included if they fell inside parks or within 50 km of park boundaries. Species data retrieved from USGS Nonindigenous Aquatic Species Database (accessed 18 August 2023).

As of 2023, non-native crayfishes have been documented in 34 parks and within 50 km of an additional 259 parks (Fig. 5A, Supplementary Table 1). While the Northeast has the most species of non-native crayfishes, the Rusty (*Faxonius rusticus*), Red Swamp (*Procambarus clarkii*), and Virile Crayfish (*Faxonius virilis*) comprise most of invasive range for crayfishes, especially in the upper Midwest and West (Supplementary Table 1). Non-native amphibians have been documented in 70 parks and within 50 km of an additional 105 parks (Fig. 5B, Supplementary Table 2). American Bullfrogs comprise the largest portion of non-native amphibian records. Other notable species of conservation concern that are invasive in several states include Cuban Treefrog (*Osteopilus septentrionalis*), Cane Toad (*Rhinella marina*), African Clawed Frog (*Xenopus laevis*), Common Coqui (*Eleutherodactylus coqui*) and Greenhouse Frog (*E. planirostris*). While the non-native amphibians we assessed are most common in Florida and elsewhere along the Gulf of Mexico coast, non-native species are present in a larger ratio of parks in the Southwest than all other regions except for the Pacific Islands, which lack native amphibians (Figs. 2 and 5^20^).

**Fig. 6:**
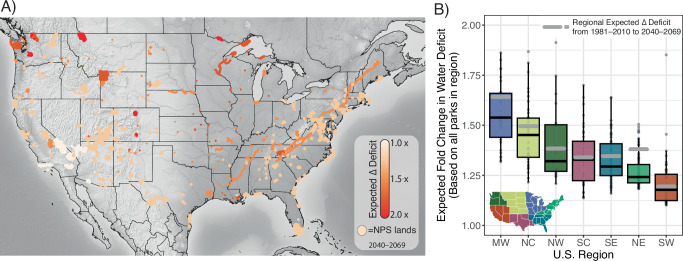
Forecasted mid-century (2040‒2069) increases in climatic water deficit for each national park in the conterminous U.S.A. Forecasted changes in climatic water deficit are summarized relative to 1981‒2010 **A** and increases in water deficit summarized by region **B**^53^^,^^68^. The gray line in B is the average expected change in water deficit for the entire region, while the box plots represent the deficit values for all parks in that respective region. Each box encompasses the first through third quantile of the ensemble average value of all parks. Water deficit was based on precipitation inputs from 800-m, daily data aggregated into 30-year averages of annual totals. Projections were made at 1 km^2^ for the entirety of each park based on the mean of outputs from 25 climate futures for RCP4.5 and RCP8.5 climate models (see Supplementary Table 3 for details).

Projected increases in climatic water deficit from 1981‒2010 to 2040‒2069, based on an ensemble average of 25 climate futures, ranged from an average increase of 20% for parks in the Southwest to 56% for parks in the Midwest (Fig. 6A; see Methods for details). Across all parks in the conterminous U.S. (i.e., excluding Alaska, Hawaii, and island territories), the ensemble-averaged climatic water deficit was projected to increase an average of 34%, with a range of 10% in Joshua Tree National Park to 212% in Rocky Mountain National Park (Supplementary Fig. 1). The range of projected changes for parks was similar for the Midwest, North Central, Northwest, and South Central regions, whereas the least variation in projected changes were for parks in the Northeast (Fig. 6B). In all seven regions, the mean projected change in climatic water deficit for parks was lower than that expected for all lands in each respective region (Fig. 6B).

## Discussion

Globally, protected areas like national parks and other preserves have a crucial role in protecting biodiversity, especially in the face of growing threats^7,8,38^. Yet, less is known about the role of NPS lands in preserving amphibian diversity compared to most other vertebrate classes. Our characterization of patterns of rarity, species-area relationships, potential threats from non-native species, and projected increases in dryness for national parks reveal a wide range of gaps in species protection and wide range of threats, but also opportunities for targeted expansion and active management.

National parks support approximately 65% of amphibian diversity in the U.S.^20^. Species richness in parks generally reflects national patterns in warmth and moisture and the phylogeographic history of amphibians in North America^39,40^, with the greatest richness in the southeast and northeast U.S. (Fig. 2). Regions with more species of salamanders than frogs and toads (NE, NW, SE) also tended to have the steepest species–area relationships (Fig. 4). Many salamanders in these regions can persist in small habitat patches and are less dependent on surface waters than frogs and toads. However, species characterized as secure are overrepresented in parks relative to totals nationally. Globally and in the U.S., the number of amphibian species categorized as at-risk are increasing^41^. And between 2002 and 2011, declines of at-risk species in the U.S. outpaced declines for species listed as least concern, with populations in national parks declining more than those on other federal lands^13^.

Given the importance of protected lands to conservation, there is concern these lands remain absent from areas where they could best contribute to protect biodiversity^42^. At-risk amphibian species occur across the U.S. National Park System, although they comprise the greatest fraction of native species in the Southwest, Southeast, and Northwest. No species-level extinctions have been documented for amphibians that occurred in parks, but local extirpations from parks have occurred. For example, several ranid frog species have been extirpated or suffered severe declines in both small and large national parks in the western U.S., often in response to invasive species, disease, or climate-induced changes to habitat^14–16,18^.

Many at-risk amphibians also have ranges that include several national parks, yet the species have not been documented in parks; these species are limited almost exclusively to the southern half of the U.S. (Fig. 3B). The range of the federally threatened Chiricahua Leopard Frog (*Lithobates chiricahuensis*) encompasses several small national parks in the Southwest and South Central regions, but the distribution of this species is fragmented and many parks in the region have limited aquatic habitats^18,43^. Similarly, some at-risk amphibian species in the Southeast, such as the Frosted Flatwoods Salamander (*Ambystoma cingulatum*), Ornate Chorus Frog (*Pseudacris ornata*), and Gopher Frog (*Lithobates capito*), had large historical ranges that overlapped several parks, but these species currently occupy only a small portion of their historical ranges or parks do not provide suitable habitat^44,45^. By comparison, 54% of native amphibians had been documented on Department of Defense lands in the continental U.S. as of 2017^46^. These lands included critical habitat for some at-risk species not documented in parks, including for all the species mentioned earlier in this paragraph^46^. These differences illustrate the potential for complementary roles of different classes of protected lands in safeguarding biodiversity against threats.

Based on our summaries, non-native crayfishes occur in less than 10% of national parks and non-native amphibians occur in at least 16% of parks (Fig. 5, Supplementary Tables 1, 2). But when we include small watersheds within 50 km of park boundaries, these numbers swell to 60% and 25% of parks threatened by non-native crayfish and amphibians, respectively. The Rusty Crayfish (*Orconectes rusticus*), in particular, has invaded a wide range of waterbodies and national parks in the upper Midwest^47^ (Fig. 5A). In the West, Red Swamp Crayfish (*Procambarus clarkii*) sometimes displace amphibians^48^, and in Crater Lake National Park (Oregon), introduced Signal Crayfish (*Pacifastacus leniusculus*) threaten the persistence of a distinct population of Rough-skinned Newts (*Taricha granulosa*)^49^. While it is clear non-native crayfish can sometimes limit amphibian populations, the geographic scope of these threats remains unclear, in part because non-native crayfishes, fishes, and amphibians tend to co-occur or become common in disturbed habitats^28,48^.

Non-native amphibians are associated with the local or regional decline of several native amphibians, including on national park lands^14,34^. Most large national parks in the western U.S. are at high elevations or have cold winters. These features have provided thermal refugia for some native species while also limiting spread of most non-native amphibians, but continued warming is likely to aid invasion by non-native amphibians that come from warm regions^25,26^. Historically, non-native predators have been less of a conservation threat to amphibians in the eastern U.S., but non-native Cuban Treefrogs have displaced native treefrogs in Everglades National Park and Big Cypress National Preserve^14^. Non-native fishes are also important predators linked with declines of several at-risk amphibians, including in national parks^17,19^. We did not quantify the overlap between amphibians and non-native fishes because fishes are still often managed as a source of recreation, which highlights the dual, and sometimes conflicting, mandates for conservation and visitation in U.S. national parks^50^.

Changes in temperature and precipitation could amplify effects of other stressors (e.g., invasive species) and contribute to population declines and range restrictions of both at-risk and common species. Like most areas, U.S. national parks are overwhelmingly warmer than they were historically^51^, with mean annual temperatures across all parks increasing ~1.0 °C over the last century^52^. Precipitation between 1895 and 2010 declined in national parks overall, especially in Alaska (~7% decline) and the Pacific Islands (~4% decline). In the conterminous U.S., parks in the already arid Southwest became drier^52,53^. Across all parks in the conterminous U.S., our ensemble-averaged approach produced a 34% increase in projected, annual climatic water deficit by mid-century (1981–2010 vs. 2040–2069; Fig. 6, Supplementary Fig. 1). Although there was a wide range of projections within regions and among climate scenarios, the largest increases were for the Midwest and North Central and the smallest increases were for the Southwest and Northeast. The Midwest and North Central regions are dominated by amphibian species that are habitat generalists and have large ranges. We suspect these generalist species will be less sensitive to climate changes than endemics with small ranges, such as the Shenandoah Salamander and Jemez Mountains Salamander, many of which are already limited to small patches of high-elevation habitat that are threatened by drying.

Despite projected increases in climatic water deficit, many parks are expected to dry less than their surrounding regions (see gray lines in Fig. 6B). National parks are often at high elevations, near large waterbodies, or have complex topography; these features help buffer against changes or produce diverse microclimates that provide climate refugia^54,55^. Parks can also serve as areas for future colonization or conservation translocations, especially for species with range edges nearby. In Yosemite National Park, federally threatened California Red-legged Frogs (*Rana draytonii*) were experimentally translocated to provide refuge against invasive American Bullfrogs and climate changes at lower elevations^34^. In some regions, climate changes may also open areas not previously available to amphibians (e.g., refs. ^56–58^), which could partially offset losses due to warming or drying.

Although National Park Service lands are protected, there are still management opportunities such as restoring or protecting critical habitats, including eradicating non-native species^19,49,59^; implementing biosecurity and early detection and rapid responses to threats^31^; and reactive or proactive translocations^14,34^. Area-based expansion is also essential to achieving national and global conservation goals^1,10^, and for the NPS, can help protect underrepresented ecosystems or natural resources such as freshwater biodiversity hotspots and grasslands^35^. Targeted expansion to reduce these shortfalls could help protect habitat for several at-risk amphibians (e.g., Fig. 3) and other species. The species–area relationships we described (Fig. 4) show that in some regions of the U.S. (e.g., Northeast and Southeast), there is a strong potential for biodiversity benefits with even modest expansions to existing parks.

Maintaining biodiversity rests heavily on formal and informal protection of whole landscapes, including lands outside of parks and preserves^60–62^. Conservation efforts to protect amphibians clearly cannot rest fully on the National Park Service or any other land management agency. Instead, partnering in regional conservation strategies can protect ecoregional biodiversity and advance natural resource stewardship^62^. Because the climatic drivers causing increased warming and drying in most areas are anticipated to intensify, the potential for parks to serve as climate refugia may also represent ideal locations for active management that is climate- and future-focused (e.g., refs. ^63,64^). Climate vulnerability assessments will also be critical for predicting shifts in species distributions or loss of critical habitat, integrating results into adaptation planning, and for identifying climate-informed opportunities and strategies for targeted conservation.

## Methods

### Occurrence data for native amphibians

To summarize species-level representation and threats to amphibians on National Park Service (NPS) lands, we used a recently updated amphibian dataset with occurrence records for 292 of 423 parks^20^. We cross-referenced species according to state-level^37^ and global conservation ranks^36^ to reflect the status of species (Fig. 1). We summarized species accounts per park based on these conservation ranks and organized output into seven geographical regions. To help inform conservation planning, including land acquisitions or management agreements, we also plotted the number of amphibian species against park size in each region^20^. We excluded Alaska, Pacific Islands, and Caribbean Islands because of low richness of native species or because large areas of Alaska have unsuitable climates for all but a very few amphibians.

### Occurrence data for non-native species

We used publicly available data to summarize threats to native amphibians based on numbers of non-native crayfish and non-native amphibian species and projected mid-century changes in climatic water deficit. Although most non-native species introduced to an area do not become invasive and may be of little conservation concern, we expect the number of non-native species reflects cumulative threats. We identified the number of non-native crayfishes and amphibians within or near (≤50 km) park boundaries based on the USGS Nonindigenous Aquatic Species database (https://nas.er.usgs.gov/). We excluded the native distributions of species indigenous to the U.S. that have spread outside of their original range (e.g., native transplants such as the Virile Crayfish and American Bullfrog).

### Climate data

To represent potential risk from climate change, we used publicly-available climate data for national parks to summarize projected water deficit during 2040‒2069^43,53^ (http://screenedcleanedsummaries.s3-website-us-west-2.amazonaws.com/). Climatic water deficit is a measure of drought that is independent of the vegetation present^65^. While amphibians use a wide range of habitats for breeding, foraging, and dormant periods, most are susceptible to changes in moisture^40^, and similar approaches based on water deficit have helped explain dynamics in wetland filling and amphibian breeding^66,67^. Water deficit was based on precipitation inputs from 800-m, daily data aggregated into 30-year averages of annual totals^53,68^. Projections were made at 1 km^2^ for the entirety of each park based on the mean of outputs from 25 climate futures for RCP4.5 and RCP8.5 climate models (Supplementary Table 3)^68^. We calculated relative change by dividing projected climate water deficit for 2040‒2069 by the 1981‒2010 baseline for each 1-km^2^ pixel, then averaging that ratio for all pixels within a park. We then used the same methods to calculate the mean climatic water deficit for the entirety of the seven regions across the conterminous U.S. Data to project changes in climatic water deficit were not available for Alaska, the Caribbean Islands, or the Pacific Islands. All climate data were downloaded from http://screenedcleanedsummaries.s3-website-us-west-2.amazonaws.com/.

## Supplementary information

Supplementary Information

## Data Availability

The amphibian data are available from the NPS DataStore at 10.57830/2301647. The data on non-native species are available from the USGS Nonindigenous Aquatic Species database (https://nas.er.usgs.gov/). All climate data are available from http://screenedcleanedsummaries.s3-website-us-west-2.amazonaws.com/.
