# Supplementary material for: Amphibian richness, rarity, threats, and conservation prospects across the U.S. National Park System
Source: NPJ Biodivers. 2024 Nov 21;3:35. doi: 10.1038/s44185-024-00067-1 (PMC11582706; doi:10.1038/s44185-024-00067-1)
Supplement: Supplementary file 1 — Supplementary Information [file 44185_2024_67_MOESM1_ESM.docx]

**Amphibian richness, rarity, threats, and conservation prospects across the U.S. National Park System**

Benjamin J. LaFrance, Andrew M. Ray, Michael T. Tercek, Robert N. Fisher, Blake R. Hossack

**Supplemental Table 1**. Non-native crayfishes documented on National Park Service (NPS) lands or within 50 km of park boundaries, based on data from the U.S. Geological Survey’s Nonindigenous Aquatic Species (NAS) database (<https://nas.er.usgs.gov/>; accessed on 2023-08-18). These summaries include all NPS lands in the USA, not just the 292 with amphibian records.

| **NPS Park Code** | **Species Documented Within Park** | **Species Documented Within 50km of Park** |
| --- | --- | --- |
| ADAM | NA | Faxonius virilis |
| AFBG | NA | Faxonius immunis, Faxonius neglectus, Faxonius obscurus, Faxonius rusticus, Faxonius virilis, Procambarus clarkii |
| ALPO | NA | Faxonius obscurus, Faxonius rusticus |
| ANDE | NA | Faxonius palmeri creolanus |
| ANJO | NA | Faxonius juvenilis, Faxonius virilis |
| ANTI | Faxonius rusticus, Faxonius virilis | Faxonius obscurus, Procambarus clarkii, Procambarus zonangulus |
| APCO | NA | Faxonius virilis, Procambarus clarkii |
| APIS | NA | Faxonius immunis, Faxonius rusticus |
| APPA | Faxonius rusticus, Faxonius virilis | Cambarus robustus, Faxonius immunis, Faxonius juvenilis, Faxonius neglectus, Faxonius obscurus, Procambarus clarkii, Procambarus zonangulus |
| ARCH | NA | Faxonius virilis |
| ARHO | NA | Faxonius rusticus, Faxonius virilis, Procambarus clarkii |
| ASIS | NA | Procambarus clarkii, Procambarus zonangulus |
| AZRU | NA | Faxonius causeyi |
| BAND | NA | Faxonius causeyi, Faxonius virilis |
| BEPA | NA | Faxonius rusticus, Faxonius virilis, Procambarus clarkii |
| BICR | NA | Faxonius virilis |
| BISO | NA | Faxonius juvenilis, Faxonius rusticus |
| BLRI | NA | Faxonius cristavarius, Faxonius juvenilis, Faxonius rusticus, Faxonius virilis, Procambarus clarkii |
| BLRV | Faxonius virilis | Cambarus robustus, Faxonius rusticus |
| BLUE | NA | Faxonius virilis |
| BOAF | NA | Faxonius virilis |
| BOHA | NA | Faxonius virilis |
| BOST | NA | Faxonius virilis |
| BOWA | NA | Faxonius virilis |
| BRCR | NA | Faxonius virilis |
| BRVB | NA | Faxonius luteus |
| BUFF | NA | Procambarus clarkii |
| CABR | NA | Procambarus clarkii |
| CACL | NA | Faxonius immunis, Faxonius neglectus, Faxonius obscurus, Faxonius rusticus, Faxonius virilis, Procambarus clarkii |
| CAHA | Procambarus clarkii | NA |
| CAJO | NA | Procambarus clarkii |
| CALO | NA | Procambarus clarkii |
| CANY | Faxonius virilis | NA |
| CARL | NA | Faxonius rusticus, Procambarus clarkii |
| CASA | NA | Procambarus clarkii |
| CATO | NA | Faxonius obscurus, Faxonius rusticus, Faxonius virilis, Procambarus clarkii |
| CAWO | NA | Faxonius rusticus, Faxonius virilis, Procambarus clarkii |
| CEBE | NA | Faxonius obscurus, Faxonius virilis, Procambarus zonangulus |
| CEBR | NA | Faxonius virilis |
| CECH | NA | Procambarus clarkii |
| CHAM | NA | Faxonius virilis |
| CHAT | NA | Procambarus clarkii |
| CHIC | Procambarus clarkii | NA |
| CHIS | NA | Procambarus clarkii |
| CHOH | Faxonius virilis | Faxonius juvenilis, Faxonius obscurus, Faxonius rusticus, Procambarus clarkii, Procambarus zonangulus |
| CHPI | NA | Procambarus clarkii |
| CHYO | NA | Procambarus clarkii |
| CIRO | NA | Faxonius virilis |
| CLBA | NA | Faxonius rusticus, Faxonius virilis, Procambarus clarkii |
| COGA | NA | Faxonius rusticus, Faxonius virilis, Procambarus clarkii |
| COLM | NA | Faxonius virilis |
| COLO | NA | Procambarus clarkii |
| CONG | NA | Procambarus clarkii |
| CORO | NA | Faxonius causeyi, Faxonius virilis, Procambarus clarkii |
| COWP | NA | Faxonius rusticus, Procambarus clarkii |
| CRLA | Pacifastacus leniusculus | Faxonius neglectus, Faxonius virilis, Procambarus clarkii |
| CRMP | NA | Faxonius virilis |
| CUGA | NA | Cambarellus shufeldtii, Faxonius juvenilis, Faxonius rusticus |
| CUIS | NA | Procambarus clarkii |
| CUVA | NA | Faxonius rusticus, Faxonius virilis, Procambarus clarkii |
| DAAV | NA | Procambarus clarkii |
| DDEM | NA | Faxonius rusticus, Faxonius virilis, Procambarus clarkii |
| DELA | NA | Faxonius rusticus, Procambarus clarkii |
| DEVA | NA | Procambarus clarkii |
| DEWA | Faxonius rusticus | Faxonius virilis, Procambarus clarkii |
| DINO | NA | Faxonius virilis |
| EBLA | NA | Faxonius sanbornii, Faxonius virilis, Procambarus clarkii |
| EDAL | NA | Faxonius obscurus, Faxonius rusticus, Faxonius virilis, Procambarus clarkii |
| EDIS | NA | Faxonius rusticus, Faxonius virilis, Procambarus clarkii |
| EFMO | NA | Faxonius rusticus |
| EISE | Faxonius rusticus, Faxonius virilis | Faxonius obscurus |
| ELMA | NA | Faxonius causeyi |
| ELMO | NA | Faxonius causeyi |
| ELRO | NA | Cambarus robustus, Faxonius neglectus, Faxonius rusticus, Faxonius virilis |
| EUON | NA | Faxonius virilis, Pacifastacus leniusculus, Procambarus clarkii |
| FEHA | NA | Faxonius immunis, Faxonius neglectus, Faxonius obscurus, Faxonius rusticus, Faxonius virilis, Procambarus clarkii |
| FIIS | NA | Procambarus clarkii |
| FILA | NA | Procambarus clarkii |
| FLNI | NA | Faxonius obscurus, Faxonius virilis |
| FOBU | NA | Faxonius virilis |
| FOCA | NA | Procambarus clarkii |
| FOFR | NA | Procambarus clarkii |
| FOMC | NA | Faxonius rusticus, Faxonius virilis, Procambarus clarkii |
| FOMR | NA | Procambarus clarkii |
| FONE | NA | Faxonius immunis, Faxonius virilis |
| FOPO | NA | Faxonius virilis, Pacifastacus leniusculus, Procambarus clarkii |
| FOPU | NA | Procambarus clarkii |
| FORA | NA | Procambarus clarkii |
| FOSC | NA | Faxonius virilis |
| FOST | NA | Faxonius obscurus, Faxonius rusticus |
| FOSU | NA | Procambarus clarkii |
| FOTH | NA | Faxonius rusticus, Faxonius virilis, Procambarus clarkii |
| FOUN | NA | Faxonius virilis |
| FOWA | NA | Faxonius rusticus, Faxonius virilis, Procambarus clarkii |
| FRDE | NA | Faxonius rusticus, Faxonius virilis, Procambarus clarkii |
| FRDO | NA | Faxonius rusticus, Faxonius virilis, Procambarus clarkii |
| FRHI | NA | Faxonius immunis |
| FRLA | NA | Faxonius virilis |
| FRRI | NA | Faxonius rusticus, Faxonius virilis |
| FRSP | NA | Faxonius virilis, Procambarus clarkii |
| FRST | NA | Faxonius obscurus, Faxonius rusticus, Faxonius virilis, Procambarus clarkii |
| GARI | NA | Faxonius virilis |
| GATE | NA | Faxonius neglectus, Faxonius rusticus, Faxonius virilis, Procambarus clarkii |
| GEGR | NA | Faxonius immunis, Faxonius neglectus, Faxonius obscurus, Faxonius rusticus, Faxonius virilis, Procambarus clarkii |
| GETT | Faxonius rusticus, Faxonius virilis | Faxonius obscurus |
| GEWA | NA | Procambarus clarkii |
| GICL | NA | Faxonius virilis |
| GLAC | NA | Faxonius virilis |
| GLCA | Faxonius causeyi | Faxonius virilis |
| GOGA | Pacifastacus leniusculus | Faxonius virilis, Procambarus clarkii |
| GOIS | NA | Faxonius immunis, Faxonius neglectus, Faxonius obscurus, Faxonius rusticus, Faxonius virilis, Procambarus clarkii |
| GRCA | NA | Faxonius causeyi, Faxonius virilis |
| GREE | NA | Faxonius rusticus, Faxonius virilis, Procambarus clarkii |
| GRPO | NA | Faxonius rusticus |
| GRSM | Procambarus clarkii | Faxonius juvenilis, Faxonius rusticus, Faxonius virilis |
| GUCO | NA | Procambarus clarkii |
| GUIS | NA | Procambarus zonangulus |
| GWCA | NA | Faxonius virilis |
| GWMP | NA | Faxonius rusticus, Faxonius virilis, Procambarus clarkii |
| HAFE | Faxonius virilis | Faxonius rusticus, Procambarus clarkii, Procambarus zonangulus |
| HAFO | NA | Faxonius virilis |
| HAGR | NA | Faxonius immunis, Faxonius neglectus, Faxonius obscurus, Faxonius rusticus, Faxonius virilis, Procambarus clarkii |
| HALE | NA | Procambarus clarkii |
| HAMP | NA | Faxonius obscurus, Faxonius rusticus, Faxonius virilis, Procambarus clarkii |
| HART | NA | Cambarus robustus, Faxonius propinquus, Faxonius rusticus |
| HATU | Procambarus clarkii | Procambarus zonangulus |
| HAVO | NA | Procambarus clarkii |
| HEHO | NA | Faxonius rusticus |
| HOBE | NA | Faxonius virilis |
| HOFR | NA | Cambarus robustus, Faxonius neglectus, Faxonius rusticus, Faxonius virilis |
| HOFU | NA | Faxonius obscurus, Faxonius rusticus, Procambarus clarkii |
| HOME | NA | Faxonius neglectus neglectus |
| HONO | NA | Procambarus clarkii |
| HOSP | NA | Faxonius virilis |
| HSTR | NA | Faxonius luteus, Faxonius virilis |
| IATR | NA | Faxonius rusticus, Procambarus clarkii |
| INDE | NA | Faxonius obscurus, Faxonius rusticus, Faxonius virilis, Procambarus clarkii |
| INDU | NA | Faxonius rusticus, Procambarus clarkii |
| ISRO | NA | Faxonius rusticus |
| JAGA | NA | Faxonius rusticus, Faxonius virilis |
| JECA | NA | Faxonius luteus |
| JEFF | NA | Procambarus clarkii |
| JEFM | NA | Faxonius rusticus, Faxonius virilis, Procambarus clarkii |
| JICA | NA | Faxonius palmeri creolanus |
| JODA | Faxonius neglectus, Faxonius rusticus | NA |
| JOFI | NA | Faxonius virilis |
| JOFL | NA | Faxonius obscurus |
| JOMU | NA | Faxonius virilis, Pacifastacus leniusculus, Procambarus clarkii |
| JOTR | NA | Procambarus clarkii |
| KAHO | NA | Procambarus clarkii |
| KALA | NA | Procambarus clarkii |
| KEMO | NA | Procambarus clarkii |
| KEWE | NA | Faxonius rusticus |
| KICA | NA | Pacifastacus leniusculus, Procambarus clarkii |
| KIMO | NA | Faxonius virilis, Procambarus clarkii |
| KLGO | NA | Faxonius virilis, Procambarus clarkii |
| KOWA | NA | Faxonius rusticus, Faxonius virilis, Procambarus clarkii |
| LACH | NA | Faxonius virilis |
| LAKE | Procambarus clarkii | Cherax quadricarinatus, Faxonius virilis |
| LARO | Faxonius virilis | NA |
| LAVO | NA | Faxonius virilis, Pacifastacus leniusculus |
| LIBO | NA | Procambarus clarkii |
| LINC | NA | Faxonius rusticus, Faxonius virilis, Procambarus clarkii |
| LIRI | NA | Faxonius rusticus, Faxonius virilis, Procambarus clarkii |
| LONG | NA | Faxonius virilis |
| LOSA | Faxonius rusticus | Faxonius immunis |
| LOWE | NA | Faxonius virilis |
| LYBA | NA | Faxonius rusticus, Faxonius virilis, Procambarus clarkii |
| MABE | NA | Faxonius rusticus, Faxonius virilis, Procambarus clarkii |
| MABI | NA | Cambarus robustus, Faxonius rusticus, Faxonius virilis |
| MALL | NA | Faxonius rusticus, Faxonius virilis, Procambarus clarkii |
| MALU | NA | Procambarus clarkii |
| MANA | NA | Faxonius virilis, Procambarus clarkii |
| MANZ | NA | Procambarus clarkii |
| MAPR | NA | Faxonius causeyi, Faxonius rusticus, Faxonius virilis |
| MAVA | NA | Cambarus robustus, Faxonius rusticus, Faxonius virilis |
| MAWA | NA | Procambarus clarkii |
| MEMY | NA | Procambarus zonangulus |
| MEVE | NA | Faxonius causeyi, Faxonius virilis |
| MIIN | NA | Faxonius virilis, Procambarus clarkii |
| MIMA | NA | Faxonius rusticus, Faxonius virilis |
| MISP | NA | Faxonius rusticus |
| MISS | Faxonius rusticus | NA |
| MLKM | NA | Faxonius rusticus, Faxonius virilis, Procambarus clarkii |
| MNRR | Faxonius rusticus | Procambarus clarkii |
| MOCA | NA | Faxonius virilis |
| MOCR | NA | Procambarus clarkii |
| MOJA | NA | Faxonius rusticus, Procambarus clarkii |
| MONO | Faxonius virilis | Faxonius rusticus, Procambarus clarkii |
| MORA | NA | Faxonius virilis, Procambarus clarkii |
| MORR | NA | Faxonius rusticus, Faxonius virilis, Procambarus clarkii |
| MORU | NA | Faxonius luteus |
| MUWO | Pacifastacus leniusculus | Procambarus clarkii |
| NABR | NA | Faxonius virilis |
| NACA | NA | Faxonius rusticus, Faxonius virilis, Procambarus clarkii |
| NATC | NA | Procambarus zonangulus |
| NATR | NA | Cambarellus shufeldtii, Faxonella clypeata, Faxonius virilis, Procambarus clarkii, Procambarus zonangulus |
| NAVA | NA | Faxonius virilis |
| NEBE | NA | Cambarus robustus, Faxonius virilis |
| NEPE | NA | Faxonius immunis, Faxonius virilis, Procambarus clarkii |
| NERI | Faxonius virilis | NA |
| NISI | NA | Procambarus clarkii |
| NOCA | NA | Faxonius virilis |
| NOCO | NA | Faxonius rusticus, Procambarus clarkii |
| OBRI | NA | Faxonius rusticus |
| OCMU | NA | Faxonius juvenilis |
| ORCA | NA | Faxonius neglectus |
| OZAR | NA | Faxonius harrisonii, Faxonius hylas, Faxonius neglectus, Faxonius neglectus chaenodactylus, Faxonius neglectus neglectus, Faxonius virilis |
| PAAL | NA | Cherax quadricarinatus, Procambarus clarkii |
| PAAV | NA | Faxonius rusticus, Faxonius virilis, Procambarus clarkii |
| PAGR | Faxonius rusticus | Faxonius immunis, Faxonius neglectus, Faxonius obscurus, Faxonius virilis, Procambarus clarkii |
| PAIS | NA | Cherax quadricarinatus, Procambarus clarkii |
| PECO | NA | Faxonius causeyi, Faxonius virilis |
| PEFO | NA | Faxonius virilis |
| PERL | NA | Procambarus clarkii |
| PETE | NA | Procambarus clarkii |
| PETR | NA | Faxonius causeyi, Faxonius deanae, Faxonius virilis |
| PEVI | Faxonius rusticus | Procambarus clarkii |
| PIMA | NA | Procambarus clarkii |
| PINN | NA | Pacifastacus leniusculus, Procambarus clarkii |
| PISC | NA | Faxonius rusticus, Faxonius virilis, Procambarus clarkii |
| POCH | NA | Faxonius virilis, Pacifastacus leniusculus, Procambarus clarkii |
| PORE | NA | Pacifastacus leniusculus, Procambarus clarkii |
| PRWI | NA | Faxonius virilis, Procambarus clarkii |
| PUHE | NA | Procambarus clarkii |
| PUHO | NA | Procambarus clarkii |
| PULL | NA | Faxonius rusticus, Procambarus clarkii |
| RABR | NA | Faxonius virilis |
| REDW | Procambarus clarkii | NA |
| REER | NA | Procambarus clarkii |
| RICH | NA | Procambarus clarkii |
| RIRA | NA | Faxonius rusticus |
| ROCR | NA | Faxonius rusticus, Faxonius virilis, Procambarus clarkii |
| RORI | NA | Faxonius virilis, Pacifastacus leniusculus, Procambarus clarkii |
| ROWI | NA | Cambarus robustus, Faxonius virilis |
| RRBH | NA | Faxonius rusticus |
| SACN | Faxonius rusticus | Faxonius immunis |
| SAFR | NA | Faxonius virilis, Pacifastacus leniusculus, Procambarus clarkii |
| SAGA | NA | Cambarus robustus, Faxonius rusticus, Faxonius virilis |
| SAGU | NA | Faxonius virilis, Procambarus clarkii |
| SAHI | NA | Cambarus robustus, Faxonius neglectus, Faxonius rusticus, Faxonius virilis, Procambarus clarkii |
| SAIR | NA | Faxonius virilis |
| SAJU | NA | Cherax quadricarinatus |
| SAMA | NA | Faxonius virilis |
| SAMO | Procambarus clarkii | Cherax quadricarinatus |
| SAPA | NA | Faxonius immunis, Faxonius neglectus, Faxonius obscurus, Faxonius rusticus, Faxonius virilis, Procambarus clarkii |
| SAPU | NA | Faxonius deanae, Faxonius rusticus, Faxonius virilis, Procambarus clarkii |
| SARA | NA | Cambarus robustus, Faxonius rusticus, Faxonius virilis |
| SEQU | NA | Procambarus clarkii |
| SHEN | NA | Faxonius obscurus, Faxonius virilis |
| SLBE | Faxonius rusticus | NA |
| SPAR | NA | Cambarus robustus, Faxonius virilis |
| STEA | NA | Faxonius obscurus, Faxonius rusticus |
| STLI | NA | Faxonius immunis, Faxonius neglectus, Faxonius obscurus, Faxonius rusticus, Faxonius virilis, Procambarus clarkii |
| STON | NA | Faxonius immunis, Faxonius neglectus, Faxonius obscurus, Faxonius rusticus, Faxonius virilis, Procambarus clarkii |
| STRI | NA | Faxonius rusticus, Faxonius virilis |
| TAPR | NA | Faxonius virilis |
| THIS | NA | Faxonius rusticus, Faxonius virilis, Procambarus clarkii |
| THKO | NA | Faxonius obscurus, Faxonius rusticus, Faxonius virilis, Procambarus clarkii |
| THRB | NA | Faxonius immunis, Faxonius neglectus, Faxonius obscurus, Faxonius rusticus, Faxonius virilis, Procambarus clarkii |
| THST | NA | Faxonius virilis, Procambarus clarkii |
| TICA | NA | Faxonius virilis |
| TIMU | NA | Procambarus clarkii |
| TONT | NA | Faxonius virilis |
| TUAI | NA | Faxonius virilis |
| TUMA | NA | Faxonius causeyi, Faxonius virilis |
| TUPE | NA | Faxonius virilis |
| TUSK | NA | Cherax quadricarinatus, Faxonius rusticus, Procambarus clarkii |
| TUZI | NA | Faxonius virilis |
| ULSG | NA | Procambarus clarkii |
| UPDE | Faxonius rusticus | NA |
| VAFO | Faxonius rusticus | Faxonius obscurus, Faxonius virilis, Procambarus clarkii |
| VALL | Faxonius virilis | Faxonius causeyi |
| VAMA | NA | Cambarus robustus, Faxonius neglectus, Faxonius rusticus, Faxonius virilis |
| VICK | NA | Faxonella clypeata |
| VIVE | NA | Faxonius rusticus, Faxonius virilis, Procambarus clarkii |
| VOYA | NA | Faxonius rusticus |
| WABA | NA | Faxonius virilis |
| WACA | NA | Faxonius virilis |
| WAMO | NA | Faxonius rusticus, Faxonius virilis, Procambarus clarkii |
| WEFA | NA | Cambarus robustus, Faxonius immunis, Faxonius neglectus, Faxonius obscurus, Faxonius rusticus, Faxonius virilis, Procambarus clarkii |
| WHHO | NA | Faxonius rusticus, Faxonius virilis, Procambarus clarkii |
| WHIS | NA | Procambarus clarkii |
| WICA | NA | Faxonius luteus |
| WICR | NA | Faxonius luteus, Faxonius virilis, Procambarus clarkii |
| WIHO | NA | Procambarus clarkii |
| WORI | NA | Faxonius rusticus |
| WOTR | NA | Faxonius rusticus, Faxonius virilis, Procambarus clarkii |
| WRBR | NA | Procambarus clarkii |
| WWII | NA | Faxonius rusticus, Faxonius virilis, Procambarus clarkii |
| WWIM | NA | Faxonius rusticus, Faxonius virilis, Procambarus clarkii |
| YOSE | NA | Faxonius virilis, Pacifastacus leniusculus, Procambarus clarkii |
| ZION | NA | Faxonius virilis |

**Supplemental Table 2.** Non-native amphibians documented on National Park Service (NPS) lands or within 50 km of park boundaries, based on data from the U.S. Geological Survey’s Nonindigenous Aquatic Species (NAS) database (<https://nas.er.usgs.gov/>; accessed on 2023-08-18). These summaries include all NPS lands in the USA, not just the 292 with amphibian records.

| **NPS Park Code** | **Species Documented Within Park** | **Species Documented Within 50km of Park** |
| --- | --- | --- |
| ADAM | NA | Cynops pyrrhogaster, Dryophytes cinereus, Lithobates catesbeianus, Osteopilus septentrionalis, Xenopus laevis |
| AFBG | NA | Osteopilus septentrionalis |
| AMIS | NA | Eleutherodactylus cystignathoides |
| APCO | NA | Dryophytes cinereus, Osteopilus septentrionalis |
| APPA | NA | Desmognathus santeetlah, Dryophytes cinereus, Dryophytes squirellus, Lithobates catesbeianus, Lithobates sphenocephalus, Necturus maculosus, Osteopilus septentrionalis |
| ARCH | Lithobates catesbeianus | NA |
| ARHO | NA | Osteopilus septentrionalis, Xenopus laevis |
| AZRU | NA | Lithobates catesbeianus |
| BADL | NA | Lithobates catesbeianus |
| BAND | NA | Lithobates catesbeianus |
| BEPA | NA | Osteopilus septentrionalis, Xenopus laevis |
| BIBE | Dryophytes cinereus, Lithobates catesbeianus | NA |
| BICY | Eleutherodactylus planirostris, Osteopilus septentrionalis | Eleutherodactylus coqui, Eleutherodactylus portoricensis, Lithobates clamitans, Ranoidea caerulea, Rhinella marina, Xenopus laevis |
| BISC | NA | Afrixalus fornasini, Agalychnis dacnicolor, Ambystoma gracile, Amphiuma tridactylum, Atelopus zeteki, Bombina orientalis, Cynops orientalis, Cynops pyrrhogaster, Duttaphrynus melanostictus, Eleutherodactylus coqui, Eleutherodactylus planirostris, Eleutherodactylus portoricensis, Hymenochirus boettgeri, Kaloula pulchra, Lithobates clamitans, Notophthalmus viridescens viridescens, Osteopilus septentrionalis, Paramesotriton labiatus, Pseudacris sierra, Ranoidea caerulea, Rhaebo blombergi, Rhinella marina, Typhlonectes natans, Xenopus laevis |
| BISO | NA | Siren intermedia |
| BITH | NA | Eleutherodactylus cystignathoides |
| BLRI | NA | Dryophytes cinereus, Dryophytes squirellus, Lithobates sphenocephalus, Osteopilus septentrionalis |
| BLRV | NA | Dryophytes cinereus, Lithobates catesbeianus, Osteopilus septentrionalis, Rhinella marina, Xenopus laevis |
| BLSC | NA | Lithobates catesbeianus |
| BOAF | NA | Cynops pyrrhogaster, Dryophytes cinereus, Lithobates catesbeianus, Osteopilus septentrionalis, Xenopus laevis |
| BOHA | NA | Cynops pyrrhogaster, Dryophytes cinereus, Lithobates catesbeianus, Osteopilus septentrionalis, Xenopus laevis |
| BOST | NA | Cynops pyrrhogaster, Dryophytes cinereus, Lithobates catesbeianus, Osteopilus septentrionalis, Xenopus laevis |
| BRVB | NA | Dryophytes cinereus |
| BUFF | NA | Dryophytes cinereus |
| BUIS | NA | Eleutherodactylus coqui, Osteopilus septentrionalis, Rhinella marina |
| CABR | NA | Ambystoma gracile, Eleutherodactylus coqui, Lithobates catesbeianus, Xenopus laevis |
| CACL | NA | Osteopilus septentrionalis |
| CACO | NA | Lithobates catesbeianus, Osteopilus septentrionalis |
| CAGR | NA | Ambystoma tigrinum, Lithobates catesbeianus |
| CAMO | NA | Lithobates catesbeianus |
| CANA | NA | Eleutherodactylus planirostris, Osteopilus septentrionalis, Rhinella marina, Xenopus laevis |
| CANY | NA | Lithobates catesbeianus |
| CARL | NA | Dryophytes cinereus, Dryophytes squirellus, Lithobates sphenocephalus, Osteopilus septentrionalis |
| CASA | NA | Eleutherodactylus planirostris, Osteopilus septentrionalis |
| CAVE | Lithobates catesbeianus | NA |
| CAVO | NA | Lithobates catesbeianus |
| CAWO | NA | Osteopilus septentrionalis, Xenopus laevis |
| CECH | NA | Ambystoma tigrinum, Lithobates catesbeianus, Lithobates pipiens, Xenopus laevis |
| CHAM | NA | Lithobates catesbeianus |
| CHAT | NA | Osteopilus septentrionalis |
| CHIR | NA | Ambystoma tigrinum, Lithobates catesbeianus |
| CHIS | NA | Ambystoma gracile, Xenopus laevis |
| CHOH | NA | Osteopilus septentrionalis, Xenopus laevis |
| CHPI | NA | Eleutherodactylus planirostris, Osteopilus septentrionalis |
| CHRI | NA | Rhinella marina |
| CLBA | NA | Osteopilus septentrionalis, Xenopus laevis |
| COGA | NA | Osteopilus septentrionalis, Xenopus laevis |
| COLM | NA | Lithobates catesbeianus, Pseudacris hypochondriaca |
| CONG | NA | Dryophytes cinereus, Dryophytes gratiosus, Dryophytes squirellus |
| CORO | NA | Lithobates catesbeianus |
| CRLA | NA | Lithobates catesbeianus |
| CUGA | NA | Ambystoma tigrinum |
| CUIS | NA | Eleutherodactylus planirostris, Osteopilus septentrionalis, Rhinella marina |
| CURE | NA | Lithobates catesbeianus |
| CUVA | NA | Osteopilus septentrionalis |
| DDEM | NA | Osteopilus septentrionalis, Xenopus laevis |
| DESO | NA | Eleutherodactylus planirostris, Osteopilus septentrionalis, Pseudacris sierra, Rhinella marina, Xenopus laevis |
| DEVA | NA | Lithobates catesbeianus |
| DRTO | Eleutherodactylus planirostris | Osteopilus septentrionalis, Rhinella marina |
| EBLA | NA | Lithobates catesbeianus |
| EDAL | NA | Dryophytes gratiosus, Osteopilus septentrionalis |
| EDIS | NA | Osteopilus septentrionalis |
| EUON | NA | Bombina orientalis, Dryophytes cinereus, Lithobates berlandieri, Lithobates catesbeianus, Lithobates pipiens, Siren intermedia, Xenopus laevis |
| EVER | Eleutherodactylus planirostris, Osteopilus septentrionalis, Rhinella marina | Afrixalus fornasini, Agalychnis dacnicolor, Amphiuma tridactylum, Atelopus zeteki, Bombina orientalis, Cynops orientalis, Cynops pyrrhogaster, Duttaphrynus melanostictus, Eleutherodactylus coqui, Eleutherodactylus portoricensis, Hymenochirus boettgeri, Kaloula pulchra, Lithobates clamitans, Notophthalmus viridescens viridescens, Paramesotriton labiatus, Pseudacris sierra, Ranoidea caerulea, Rhaebo blombergi, Typhlonectes natans, Xenopus laevis |
| FEHA | NA | Osteopilus septentrionalis |
| FIIS | NA | Osteopilus septentrionalis |
| FLFO | NA | Lithobates catesbeianus, Osteopilus septentrionalis |
| FOBO | NA | Ambystoma tigrinum, Lithobates catesbeianus |
| FOCA | NA | Eleutherodactylus planirostris, Osteopilus septentrionalis, Rhinella marina |
| FODA | NA | Lithobates catesbeianus |
| FOFR | NA | Eleutherodactylus planirostris, Osteopilus septentrionalis |
| FOLS | NA | Lithobates catesbeianus |
| FOMA | Eleutherodactylus planirostris, Osteopilus septentrionalis | NA |
| FOMC | NA | Osteopilus septentrionalis |
| FOPO | NA | Bombina orientalis, Dryophytes cinereus, Lithobates berlandieri, Lithobates catesbeianus, Lithobates pipiens, Siren intermedia, Xenopus laevis |
| FOPU | NA | Eleutherodactylus planirostris, Osteopilus septentrionalis |
| FOSU | NA | Eleutherodactylus planirostris, Osteopilus septentrionalis |
| FOTH | NA | Osteopilus septentrionalis, Xenopus laevis |
| FOUN | NA | Lithobates catesbeianus |
| FOVA | NA | Lithobates catesbeianus, Osteopilus septentrionalis |
| FOWA | NA | Osteopilus septentrionalis, Xenopus laevis |
| FRDE | NA | Osteopilus septentrionalis, Xenopus laevis |
| FRDO | NA | Osteopilus septentrionalis, Xenopus laevis |
| FRLA | NA | Cynops pyrrhogaster, Dryophytes cinereus, Lithobates catesbeianus, Osteopilus septentrionalis, Xenopus laevis |
| FRSP | NA | Osteopilus septentrionalis, Xenopus laevis |
| FRST | NA | Osteopilus septentrionalis |
| GATE | NA | Osteopilus septentrionalis |
| GEGR | NA | Osteopilus septentrionalis |
| GETT | NA | Osteopilus septentrionalis |
| GICL | Lithobates catesbeianus | NA |
| GLAC | NA | Lithobates catesbeianus |
| GLBA | NA | Rana aurora |
| GLCA | Lithobates catesbeianus | NA |
| GOGA | Bombina orientalis | Dryophytes cinereus, Lithobates berlandieri, Lithobates catesbeianus, Lithobates pipiens, Siren intermedia, Xenopus laevis |
| GOIS | NA | Osteopilus septentrionalis |
| GRCA | NA | Lithobates catesbeianus |
| GREE | NA | Osteopilus septentrionalis, Xenopus laevis |
| GRSA | NA | Lithobates catesbeianus |
| GRSM | Dryophytes squirellus, Osteopilus septentrionalis | Ambystoma tigrinum, Desmognathus santeetlah |
| GRTE | Lithobates catesbeianus | NA |
| GUCO | NA | Dryophytes cinereus |
| GUIS | Eleutherodactylus planirostris, Osteopilus septentrionalis | NA |
| GUMO | NA | Lithobates catesbeianus |
| GWMP | NA | Osteopilus septentrionalis, Xenopus laevis |
| HAFO | NA | Lithobates catesbeianus |
| HAGR | NA | Osteopilus septentrionalis |
| HALE | NA | Dendrobates auratus, Eleutherodactylus coqui, Eleutherodactylus planirostris, Glandirana rugosa, Lithobates catesbeianus, Rhinella marina |
| HAMP | NA | Osteopilus septentrionalis |
| HAVO | Eleutherodactylus coqui | Eleutherodactylus planirostris, Glandirana rugosa, Lithobates catesbeianus, Rhinella marina |
| HOBE | NA | Osteopilus septentrionalis |
| HOFU | NA | Osteopilus septentrionalis |
| HOME | NA | Osteopilus septentrionalis |
| HONO | NA | Anaxyrus boreas halophilus, Dendrobates auratus, Dendrobates leucomelas, Dendrobates sp., Eleutherodactylus coqui, Eleutherodactylus planirostris, Glandirana rugosa, Lithobates catesbeianus, Lithobates clamitans, Lithobates pipiens, Pelophylax nigromaculatus, Rana aurora, Ranoidea aurea, Rhinella marina |
| HOVE | NA | Lithobates catesbeianus |
| INDE | NA | Dryophytes gratiosus, Osteopilus septentrionalis |
| INDU | NA | Osteopilus septentrionalis |
| JAGA | NA | Osteopilus septentrionalis |
| JAZZ | NA | Eleutherodactylus planirostris, Osteopilus septentrionalis |
| JEFF | NA | Osteopilus septentrionalis |
| JEFM | NA | Osteopilus septentrionalis, Xenopus laevis |
| JELA | NA | Eleutherodactylus cystignathoides, Eleutherodactylus planirostris, Osteopilus septentrionalis, Rhinella marina |
| JODA | NA | Lithobates catesbeianus |
| JODR | NA | Lithobates catesbeianus |
| JOFI | NA | Cynops pyrrhogaster, Dryophytes cinereus, Lithobates catesbeianus, Osteopilus septentrionalis, Xenopus laevis |
| JOMU | NA | Bombina orientalis, Dryophytes cinereus, Lithobates berlandieri, Lithobates catesbeianus, Lithobates pipiens, Siren intermedia, Xenopus laevis |
| JOTR | NA | Ambystoma tigrinum, Lithobates berlandieri, Lithobates catesbeianus |
| KAHO | NA | Eleutherodactylus coqui, Eleutherodactylus planirostris, Glandirana rugosa, Lithobates catesbeianus, Rhinella marina |
| KALA | Rhinella marina | Dendrobates auratus, Eleutherodactylus planirostris, Lithobates catesbeianus |
| KAWW | NA | Lithobates catesbeianus, Necturus maculosus |
| KEMO | NA | Osteopilus septentrionalis |
| KICA | NA | Lithobates catesbeianus, Lithobates pipiens |
| KIMO | NA | Dryophytes cinereus, Lithobates sphenocephalus |
| KLGO | NA | Batrachoseps attenuatus, Lithobates catesbeianus, Xenopus laevis |
| KOWA | NA | Osteopilus septentrionalis, Xenopus laevis |
| LABE | NA | Lithobates catesbeianus |
| LAKE | Lithobates catesbeianus | Ambystoma tigrinum |
| LAVO | NA | Ambystoma tigrinum, Lithobates catesbeianus, Lithobates pipiens |
| LEWI | NA | Lithobates catesbeianus |
| LINC | NA | Osteopilus septentrionalis, Xenopus laevis |
| LONG | NA | Cynops pyrrhogaster, Dryophytes cinereus, Lithobates catesbeianus, Osteopilus septentrionalis, Xenopus laevis |
| LOSA | NA | Lithobates catesbeianus |
| LOWE | NA | Cynops pyrrhogaster, Dryophytes cinereus, Lithobates catesbeianus, Osteopilus septentrionalis, Rhinella marina, Xenopus laevis |
| LYBA | NA | Osteopilus septentrionalis, Xenopus laevis |
| MABE | NA | Osteopilus septentrionalis, Xenopus laevis |
| MABI | NA | Necturus maculosus |
| MALL | NA | Osteopilus septentrionalis, Xenopus laevis |
| MALU | NA | Osteopilus septentrionalis |
| MANA | NA | Osteopilus septentrionalis, Xenopus laevis |
| MANZ | NA | Lithobates catesbeianus |
| MAPR | NA | Lithobates catesbeianus, Siren intermedia |
| MAVA | NA | Lithobates sphenocephalus |
| MEVE | NA | Lithobates catesbeianus |
| MIIN | NA | Batrachoseps attenuatus, Lithobates catesbeianus, Xenopus laevis |
| MIMA | NA | Cynops pyrrhogaster, Dryophytes cinereus, Lithobates catesbeianus, Osteopilus septentrionalis, Rhinella marina, Xenopus laevis |
| MIMI | NA | Lithobates catesbeianus |
| MISS | NA | Lithobates catesbeianus |
| MLKM | NA | Osteopilus septentrionalis, Xenopus laevis |
| MNRR | NA | Lithobates catesbeianus |
| MOCA | Lithobates catesbeianus | Lithobates clamitans, Pseudacris regilla |
| MOJA | NA | Ambystoma tigrinum, Lithobates catesbeianus |
| MORA | NA | Lithobates catesbeianus, Xenopus laevis |
| MORR | NA | Osteopilus septentrionalis |
| MORU | NA | Lithobates catesbeianus |
| MUWO | NA | Bombina orientalis, Dryophytes cinereus, Lithobates berlandieri, Lithobates catesbeianus, Lithobates pipiens, Siren intermedia, Xenopus laevis |
| NABR | NA | Lithobates catesbeianus |
| NACA | NA | Osteopilus septentrionalis, Xenopus laevis |
| NATR | NA | Eleutherodactylus planirostris |
| NEBE | NA | Cynops pyrrhogaster, Lithobates catesbeianus |
| NEPE | NA | Lithobates catesbeianus, Taricha granulosa |
| NIOB | Lithobates catesbeianus | NA |
| NISI | Dryophytes cinereus, Dryophytes gratiosus, Dryophytes squirellus | Osteopilus septentrionalis |
| NPSA | NA | Rhinella marina |
| OBRI | NA | Siren intermedia |
| OCMU | NA | Desmognathus quadramaculatus, Osteopilus septentrionalis |
| OLYM | NA | Lithobates catesbeianus |
| ORCA | NA | Lithobates catesbeianus |
| PAAL | NA | Dryophytes cinereus, Osteopilus septentrionalis |
| PAAV | NA | Osteopilus septentrionalis, Xenopus laevis |
| PAGR | NA | Osteopilus septentrionalis |
| PAIS | NA | Eleutherodactylus campi, Lithobates catesbeianus |
| PECO | NA | Lithobates catesbeianus |
| PEFO | NA | Lithobates catesbeianus |
| PERI | NA | Osteopilus septentrionalis |
| PERL | NA | Anaxyrus boreas halophilus, Dendrobates auratus, Dendrobates leucomelas, Dendrobates sp., Eleutherodactylus coqui, Eleutherodactylus planirostris, Glandirana rugosa, Lithobates catesbeianus, Lithobates clamitans, Lithobates pipiens, Pelophylax nigromaculatus, Rana aurora, Ranoidea aurea, Rhinella marina |
| PETR | NA | Lithobates catesbeianus |
| PEVI | NA | Lithobates catesbeianus |
| PIMA | NA | Ambystoma tigrinum, Lithobates berlandieri, Lithobates catesbeianus |
| PINN | NA | Lithobates catesbeianus |
| PISC | NA | Osteopilus septentrionalis, Xenopus laevis |
| POCH | NA | Bombina orientalis, Dryophytes cinereus, Lithobates berlandieri, Lithobates catesbeianus |
| PORE | NA | Bombina orientalis, Dryophytes cinereus, Lithobates berlandieri, Lithobates catesbeianus, Lithobates pipiens, Xenopus laevis |
| PRWI | NA | Osteopilus septentrionalis, Xenopus laevis |
| PUHE | NA | Eleutherodactylus coqui, Eleutherodactylus planirostris, Glandirana rugosa, Lithobates catesbeianus, Rhinella marina |
| PUHO | NA | Eleutherodactylus coqui, Eleutherodactylus planirostris, Glandirana rugosa, Lithobates catesbeianus, Rhinella marina |
| PULL | NA | Osteopilus septentrionalis |
| REDW | NA | Lithobates catesbeianus |
| REER | NA | Osteopilus septentrionalis |
| RIGR | Dryophytes cinereus, Lithobates catesbeianus | NA |
| ROCR | NA | Osteopilus septentrionalis, Xenopus laevis |
| ROMO | NA | Acris crepitans, Lithobates catesbeianus, Pseudacris hypochondriaca, Xenopus laevis |
| RORI | NA | Bombina orientalis, Dryophytes cinereus, Lithobates berlandieri, Lithobates catesbeianus, Lithobates pipiens, Siren intermedia, Xenopus laevis |
| ROWI | NA | Dryophytes cinereus, Osteopilus septentrionalis |
| SAAN | NA | Eleutherodactylus campi, Eleutherodactylus cystignathoides, Lithobates catesbeianus, Osteopilus septentrionalis |
| SACN | NA | Lithobates catesbeianus |
| SAFR | NA | Bombina orientalis, Dryophytes cinereus, Lithobates berlandieri, Lithobates catesbeianus, Lithobates pipiens, Siren intermedia, Xenopus laevis |
| SAGA | NA | Necturus maculosus |
| SAGU | Lithobates catesbeianus | Ambystoma tigrinum, Xenopus laevis |
| SAHI | NA | Osteopilus septentrionalis |
| SAIR | NA | Cynops pyrrhogaster, Dryophytes cinereus, Lithobates catesbeianus, Osteopilus septentrionalis, Xenopus laevis |
| SAJH | NA | Lithobates catesbeianus |
| SAJU | NA | Dryophytes cinereus, Lithobates catesbeianus, Lithobates grylio, Osteopilus septentrionalis, Pipa pipa, Rhinella marina, Scinax ruber |
| SAMA | NA | Cynops pyrrhogaster, Dryophytes cinereus, Osteopilus septentrionalis |
| SAMO | Lithobates catesbeianus | Eleutherodactylus coqui, Xenopus laevis |
| SAPA | NA | Osteopilus septentrionalis |
| SAPU | NA | Lithobates catesbeianus |
| SARI | NA | Rhinella marina |
| SCBL | NA | Lithobates catesbeianus |
| SEQU | NA | Lithobates catesbeianus, Lithobates pipiens |
| SHEN | NA | Osteopilus septentrionalis |
| SITK | NA | Taricha granulosa |
| SPAR | NA | Lithobates sphenocephalus, Necturus maculosus, Osteopilus septentrionalis |
| STLI | NA | Osteopilus septentrionalis |
| STON | NA | Osteopilus septentrionalis |
| TAPR | NA | Dryophytes squirellus |
| THIS | NA | Osteopilus septentrionalis, Xenopus laevis |
| THKO | NA | Dryophytes gratiosus, Osteopilus septentrionalis |
| THRB | NA | Osteopilus septentrionalis |
| THST | NA | Osteopilus septentrionalis, Xenopus laevis |
| TICA | NA | Osteopilus septentrionalis |
| TIMU | Eleutherodactylus planirostris | Osteopilus septentrionalis, Rhinella marina |
| TONT | NA | Lithobates catesbeianus |
| TUAI | NA | Osteopilus septentrionalis |
| TUIN | NA | Osteopilus septentrionalis |
| TULE | NA | Lithobates catesbeianus |
| TUMA | NA | Ambystoma tigrinum, Lithobates catesbeianus |
| TUSK | NA | Ambystoma tigrinum, Lithobates catesbeianus |
| TUZI | NA | Lithobates catesbeianus |
| ULSG | NA | Osteopilus septentrionalis |
| VAFO | NA | Osteopilus septentrionalis |
| VALL | NA | Lithobates catesbeianus |
| VICR | NA | Eleutherodactylus coqui, Osteopilus septentrionalis, Rhinella marina |
| VIIS | Osteopilus septentrionalis | Eleutherodactylus coqui, Rhinella marina |
| VIVE | NA | Osteopilus septentrionalis, Xenopus laevis |
| WACA | NA | Lithobates catesbeianus |
| WAMO | NA | Osteopilus septentrionalis, Xenopus laevis |
| WAPA | NA | Eleutherodactylus coqui, Eleutherodactylus planirostris, Fejervarya cancrivora, Fejervarya limnocharis, Litoria fallax, Microhyla pulchra, Pelophylax nigromaculatus, Polypedates megacephalus, Rhinella marina, Sylvirana guentheri |
| WHHO | NA | Osteopilus septentrionalis, Xenopus laevis |
| WHIS | NA | Lithobates catesbeianus, Lithobates pipiens |
| WHSA | NA | Lithobates catesbeianus |
| WICA | NA | Lithobates catesbeianus |
| WOTR | NA | Osteopilus septentrionalis, Xenopus laevis |
| WWII | NA | Osteopilus septentrionalis, Xenopus laevis |
| WWIM | NA | Osteopilus septentrionalis, Xenopus laevis |
| YELL | NA | Lithobates catesbeianus |
| YOSE | NA | Ambystoma tigrinum, Lithobates berlandieri, Lithobates catesbeianus, Xenopus laevis |
| YUHO | NA | Lithobates catesbeianus |

**Supplemental Table 3.** List of the 25 projected climate futures used to characterize climatic threat for amphibians on U.S. National Park Service lands. Forecasted mid-century (2040‒2069) increases in climatic water deficit for each park, relative to 1981‒2010 (A) and increases in water deficit summarized by region (B) . Water deficit was based on precipitation inputs from an 800-m, daily data aggregated into 30-year averages of annual totals. All climate data are available from [http://screenedcleanedsummaries.s3-website-us-west-2.amazonaws.com/](https://gcc02.safelinks.protection.outlook.com/?url=http%3A%2F%2Fscreenedcleanedsummaries.s3-website-us-west-2.amazonaws.com%2F&data=05%7C02%7Cblake_hossack%40usgs.gov%7Cc837f964304248b68f2408dc874c660d%7C0693b5ba4b184d7b9341f32f400a5494%7C0%7C0%7C638534009399046199%7CUnknown%7CTWFpbGZsb3d8eyJWIjoiMC4wLjAwMDAiLCJQIjoiV2luMzIiLCJBTiI6Ik1haWwiLCJXVCI6Mn0%3D%7C0%7C%7C%7C&sdata=l965BvlPyfk%2F%2Ftr9oHDvmOrykf3kDmnwUe0nVOPilRE%3D&reserved=0).

| **Model** | **RCP Level** | **Metric** | **Start Year** | **End Year** | **Period** |
| --- | --- | --- | --- | --- | --- |
| GridMET | historical data | Climatic Water Deficit | 1981 | 2010 | annual |
| BNU-ESM | rcp45 | Climatic Water Deficit | 2040 | 2069 | annual |
| CCSM4 | rcp45 | Climatic Water Deficit | 2040 | 2069 | annual |
| CNRM-CM5 | rcp45 | Climatic Water Deficit | 2040 | 2069 | annual |
| CSIRO-Mk3-6-0 | rcp45 | Climatic Water Deficit | 2040 | 2069 | annual |
| CanESM2 | rcp45 | Climatic Water Deficit | 2040 | 2069 | annual |
| GFDL-ESM2G | rcp45 | Climatic Water Deficit | 2040 | 2069 | annual |
| HadGEM2-CC365 | rcp45 | Climatic Water Deficit | 2040 | 2069 | annual |
| IPSL-CM5A-LR | rcp45 | Climatic Water Deficit | 2040 | 2069 | annual |
| MIROC5 | rcp45 | Climatic Water Deficit | 2040 | 2069 | annual |
| MRI-CGCM3 | rcp45 | Climatic Water Deficit | 2040 | 2069 | annual |
| NorESM1-M | rcp45 | Climatic Water Deficit | 2040 | 2069 | annual |
| inmcm4 | rcp45 | Climatic Water Deficit | 2040 | 2069 | annual |
| BNU-ESM | rcp85 | Climatic Water Deficit | 2040 | 2069 | annual |
| CCSM4 | rcp85 | Climatic Water Deficit | 2040 | 2069 | annual |
| CNRM-CM5 | rcp85 | Climatic Water Deficit | 2040 | 2069 | annual |
| CSIRO-Mk3-6-0 | rcp85 | Climatic Water Deficit | 2040 | 2069 | annual |
| CanESM2 | rcp85 | Climatic Water Deficit | 2040 | 2069 | annual |
| GFDL-ESM2G | rcp85 | Climatic Water Deficit | 2040 | 2069 | annual |
| HadGEM2-CC365 | rcp85 | Climatic Water Deficit | 2040 | 2069 | annual |
| IPSL-CM5A-LR | rcp85 | Climatic Water Deficit | 2040 | 2069 | annual |
| MIROC-ESM-CHEM | rcp85 | Climatic Water Deficit | 2040 | 2069 | annual |
| MIROC5 | rcp85 | Climatic Water Deficit | 2040 | 2069 | annual |
| MRI-CGCM3 | rcp85 | Climatic Water Deficit | 2040 | 2069 | annual |
| NorESM1-M | rcp85 | Climatic Water Deficit | 2040 | 2069 | annual |
| inmcm4 | rcp85 | Climatic Water Deficit | 2040 | 2069 | annual |

**Supplemental Figure 1.** Forecasted mid-century (2040‒2069) changes in climatic water deficit for U.S. national parks, relative to 1981‒2010, based on the regions shown in Figure 6 B. Each box encompasses the first through third quantile of the ensemble averaged value for 25 climate futures. Water deficit was based on precipitation inputs from an 800-m, daily data aggregated into 30-year averages of annual totals. Projections were made at 1 km*2* for the entirety of each park based on the mean of outputs from 25 climate futures for RCP4.5 and RCP8.5 climate models (see Supplemental Table 3 for details).

**
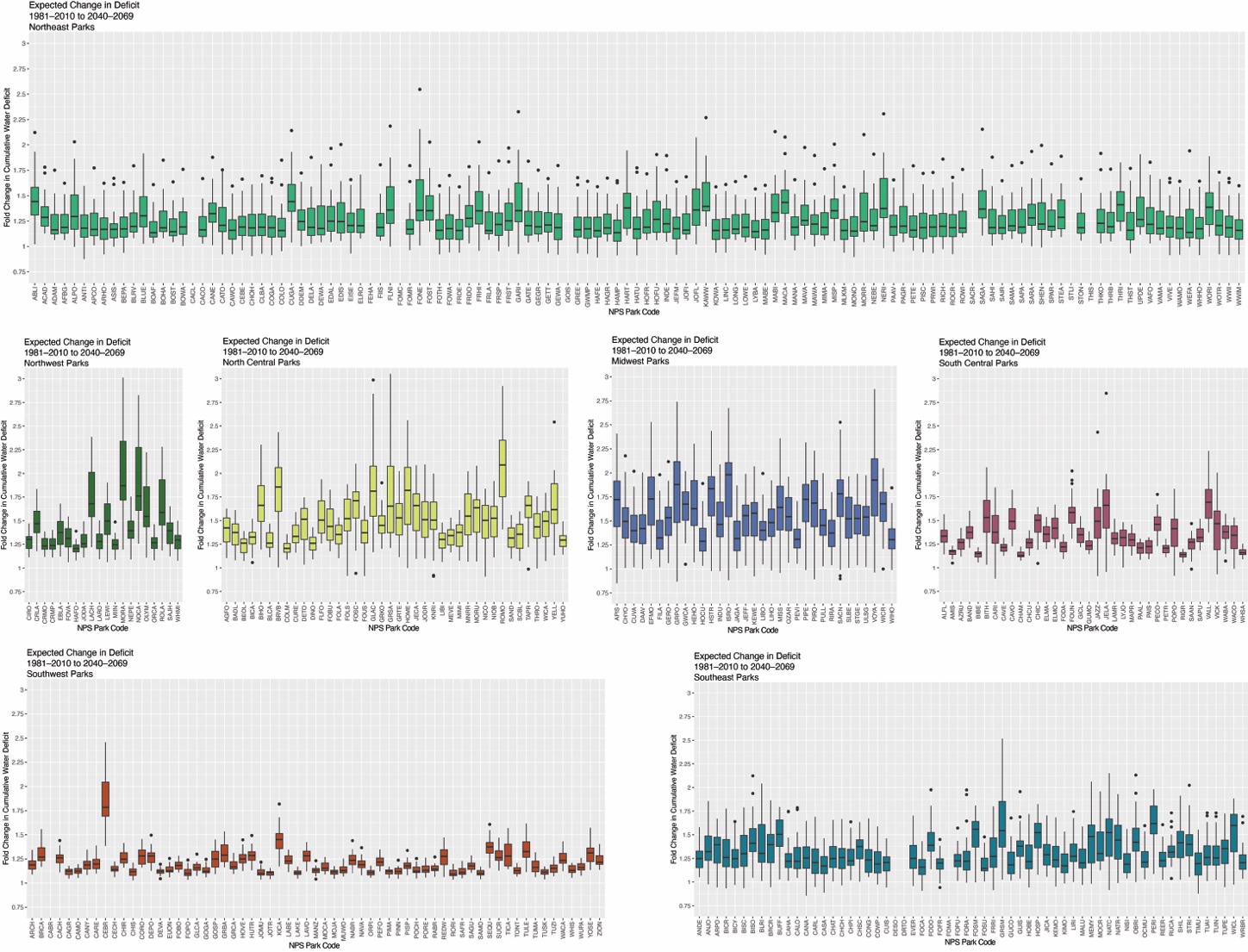
**
